# Tumor-expressed immune checkpoint B7x promotes cancer progression and antigen-specific CD8 T cell exhaustion and suppressive innate immune cells

**DOI:** 10.18632/oncotarget.21098

**Published:** 2017-09-20

**Authors:** Kim C. Ohaegbulam, Weifeng Liu, Hyungjun Jeon, Steven C. Almo, Xingxing Zang

**Affiliations:** ^1^ Department of Microbiology and Immunology, Albert Einstein College of Medicine, Bronx, NY, USA; ^2^ Department of Biochemistry, Albert Einstein College of Medicine, Bronx, NY, USA; ^3^ Department of Physiology and Biophysics, Albert Einstein College of Medicine, Bronx, NY, USA; ^4^ Department of Medicine, Albert Einstein College of Medicine, Bronx, NY, USA; ^5^ Department of Urology, Albert Einstein College of Medicine, Bronx, NY, USA

**Keywords:** B7x, immune checkpoint, pulmonary metastases, CD8 T cells, innate cells

## Abstract

B7x (B7-H4 or B7S1) is a coinhibitory member of the B7 immune checkpoint ligand family that regulates immune function following ligation with its unknown cognate receptors. B7x has limited expression on normal tissues, but is up-regulated on solid human tumors to inhibit anti-tumor immunity and associates with poor clinical prognosis. We assessed the contribution of cytokine stimuli to induce surface B7x expression on cancer cells and the role of tumor-expressed B7x in a murine pulmonary metastasis model, and finally evaluated the potential interaction between B7x and Neuropilin-1, a suggested potential cognate receptor. We showed that pro-inflammatory and anti-inflammatory cytokines IFNγ, TNFα, and IL-10 did not induce expression of B7x on human or murine cancer cells. Following i.v. injection of CT26, a murine colon cancer cell line in the BALB/c background, we observed a significant increase in tumor burden in the lung of B7x-expressing CT26 mice compared to B7x-negative parental CT26 control mice. This was marked by a significant increase in M2 tumor associated macrophages and antigen-specific CD8 T cell exhaustion. Finally, we found through multiple systems that there was no evidence for B7x and Neuropilin-1 direct interaction. Thus, the B7x pathway has an essential role in modulating the innate and adaptive immune cell infiltrate in the tumor microenvironment with its currently unknown cognate receptor(s).

## INTRODUCTION

T cell costimulation and coinhibition are mediated by proteins, notably the B7 ligand and CD28 receptor family of molecules and are critical processes regulating T cell activation, proliferation, and effector function [1, 2]. The B7 ligand and CD28 receptor family of proteins are very well characterized and has seen considerable success and interest in the field of cancer immunology due to their influence in immune evasion. Immune evasion accelerates primary tumor development and distant metastases, labeling it an important hallmark in cancer [3]. Checkpoint blockade to many of these coinhibitory proteins within this family such as cytotoxic T-lymphocyte-associated protein 4 (CTLA-4), programmed death receptor 1 (PD-1), and programmed death ligand 1 (PD-L1) have generated significant clinical therapeutic efficacy following intensive scientific inquiry into their mechanistic pathways with preclinical models [4–6]. Clearly, further studies and pathway characterization need to be pursued surrounding other less characterized members of the B7/CD28 family to achieve similar success.

B7x (B7-H4 or B7S1) is a coinhibitory B7 immune checkpoint ligand that functions to negatively modulate CD4 and CD8 T cell activity as well as the innate immune system, with limited protein expression on normal human tissues [7–11]. Various studies have shown the impact of B7x in regulating various inflammatory mediated diseases such as diabetes, bacterial infection, nephritis, EAE, and rheumatoid arthritis [12–19]. However, a majority of the studies centering on B7x have demonstrated that, in contrast to normal tissues, B7x is overexpressed on nearly all human solid tumors and associates with poor clinical outcomes [1, 2, 20, 21]. Studies utilizing B7x knockout mice have also provided evidence that host B7x contributes to increases in pulmonary metastases and primary tumor growth [22, 23]. Moreover, recent findings have concluded that blockade of tumors expressing B7x suppresses tumor growth and generates immunological memory [24, 25], supporting its therapeutic value. However, despite these recent advances, insight regarding potential induction stimuli of B7x on cancer cells and comprehensive mechanistic studies detailing its immune evasion mechanisms remain incomplete. Additionally, investigations into identifying and confirming the unknown cognate receptor(s) of B7x have stalled.

In this study, we investigated the capacity of pro-inflammatory and anti-inflammatory cytokine stimuli to induce the expression of B7x on various human and murine cancer cell lines. Also, utilizing the pulmonary experimental metastasis model with the colonic murine tumor cell line, CT26, overexpressing B7x, we revealed a detailed phenotypic description of the adaptive and innate immune cell infiltrate as a result of tumor-expressed B7x and demonstrate how it contributes to M2 macrophage polarization, along with regulatory T cell (T_regs_) and antigen-specific CD8 T cell exhaustion. Additionally, we revealed through multiple assays that B7x did not directly bind to Neuropilin-1, a recently suggested cognate receptor for B7x [26]. Collectively, our findings suggest through both the innate and adaptive arms of the immune system, the B7x pathway has a significant role in the promotion of immune evasion and carries this out through interactions with an unknown receptor(s).

## RESULTS

### B7x expression on cancer cells is unchanged following cytokine stimulation and does not affect cellular proliferation *in vitro*

**Figure 1:**
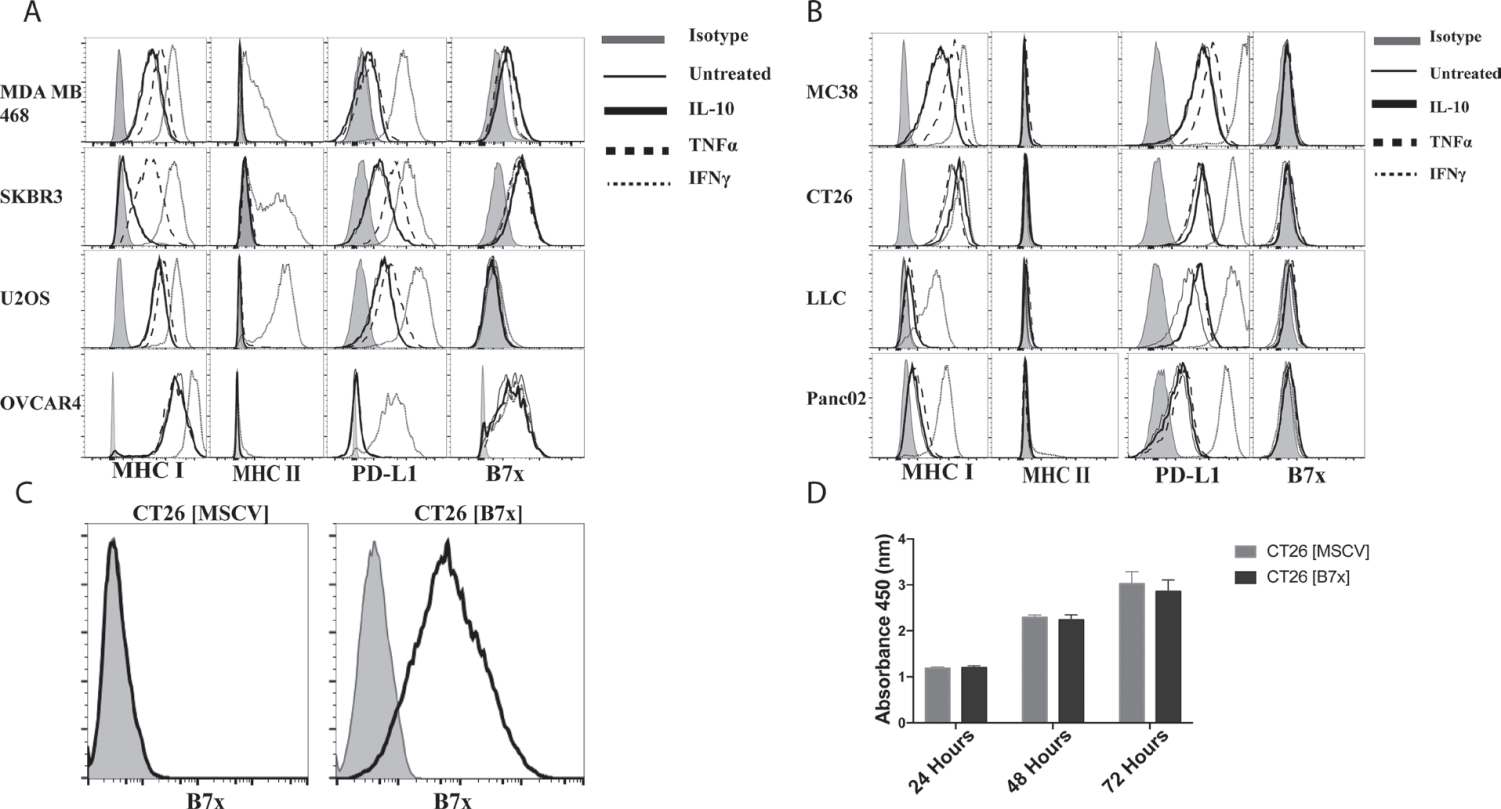
B7x expression is unaffected by cytokine stimulation and does not affect proliferation *in vitro* (**A**) Human tumor cell lines and (**B**) murine tumor cell lines were stimulated with 50 ng/mL of IL-10, TNFα, or IFNγ for 48 hours and stained for surface expression of MHC I, MHC II, PD-L1, and B7x and evaluated by FACS. (**C**) CT26 transfected with an empty MSCV retrovirus CT26 [MSCV] and CT26 transfected with a MSCV retrovirus encoding murine B7x, CT26 [B7x] were stained for B7x. (**D**) Comparison of proliferation between CT26 [MSCV] and CT26 [B7x] for 24, 48, and 72 hours with CCK-8 pooled from two independent experiments. Error bars represent SEM.

Understanding that B7x is a membranous protein that is rarely detected on normal human tissue and commonly up-regulated on the surface of cancer cells, we sought to assess the expression of B7x on a variety of human tumor cell lines and determine if common pro-inflammatory or anti-inflammatory cytokine stimulation could induce or enhance its expression. To evaluate this, we assayed four different human cancer cell lines (MDA MB 468 [breast], SKBR3 [breast], U2OS [osteosarcoma], OVCAR4 [ovary]) for their expression of endogenous B7x and stimulated each with the pro-inflammatory cytokine IFNγ, TNFα, or the anti-inflammatory cytokine IL-10 to evaluate potential change in expression following this stimulation based on current literature suggesting these cytokines regulate B7x on subsets of antigen-presenting cells (APCs) [27–30]. We observed that in the absence of stimulation, all tumor cell lines surveyed except for U2OS displayed various levels of B7x expression that did not change following cytokine stimulation. This was in sharp contrast to PD-L1, another member of the B7 ligand family, and MHC-I/II, which were significantly up-regulated to various degrees following culture with IFNγ or TNFα (Figure 1A). The same experimental approach was conducted in various murine tumor cell lines as well (MC38 [colon], CT26 [colon], LLC [lung], B16F10 [melanoma]). Though in sharp contrast to human tumor cell lines, none of the murine tumor cell lines expressed endogenous levels of B7x or enhanced expression following cytokine stimulation in the same manner as PD-L1 (Figure 1B). Thus, we concluded that IFNγ, TNFα, and IL-10 did not alter the expression of B7x in human and murine tumor cell lines.

Thus, to effectively study the role of B7x *in vivo* we engineered the colonic carcinoma cell line, CT26, derived from the BALB/c background, to stably express membranous B7x to mimic expression patterns observed in human cancer cells (Figure 1C). Furthermore, we confirmed that the expression of B7x did not cause a proliferative advantage or disadvantage to the cells *in vitro* (Figure 1D), suggesting B7x does not directly cause accelerated tumor growth independent of immune cells.

### Tumor-expressed B7x increases tumor burden in a colorectal cancer model of pulmonary metastasis

**Figure 2:**
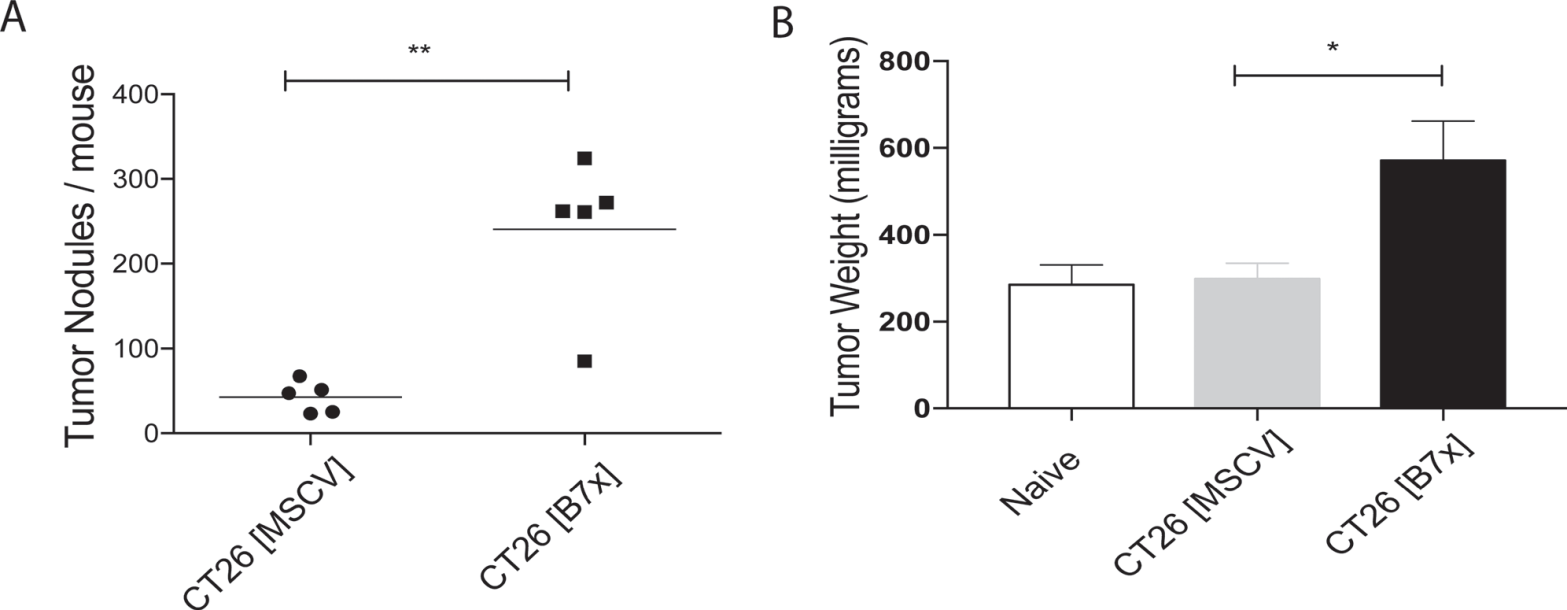
Tumor-expressed B7x increases pulmonary metastases (**A**) Quantification of lung metastases at day 17 following i.v. injection of CT26 [MSCV] or CT26 [B7x]. (**B**) Measurement of tumor weight of individual naïve, CT26 [MSCV], or CT26 [B7x] tumor bearing lungs at day 17. Data are representative of two separate experiments. *p < 0.05, **p < 0.01. Error bars represent SEM.

Wild-type mice were injected intravenously (i.v.) in the tail vein with either control CT26 cells (CT26 [MSCV]), or CT26 cells expressing stable murine B7x (CT26 [B7x]) to perform an experimental metastasis study. This standard form of tumor injection circulates the cancer cells to the heart and they largely seed in the lungs [31]. Approximately seventeen days following tumor injection we weighed the lungs and quantified the total number of metastatic tumor nodules visible on the surface of the lungs to assess tumor burden. We found that mice with tumors expressing B7x had an almost six-fold increase in the number of tumor nodules compared to the control group possessing B7x negative tumors (Figure 2A). This B7x induced increase in tumor nodule development led to a resultant significant increase in the weight of their lungs when compared to naïve mice or the CT26 control group (Figure 2B) in large part due to the additional tumor burden. Collectively this data allowed us to determine that *in vivo*, tumor expressed-B7x accelerates tumor development and increases overall tumor burden in pulmonary metastases.

### B7x promotes an increase in Foxp3^+^ Tregs and decreases proliferation and ICOS expression in antigen-specific CD8 T cells

**Figure 3:**
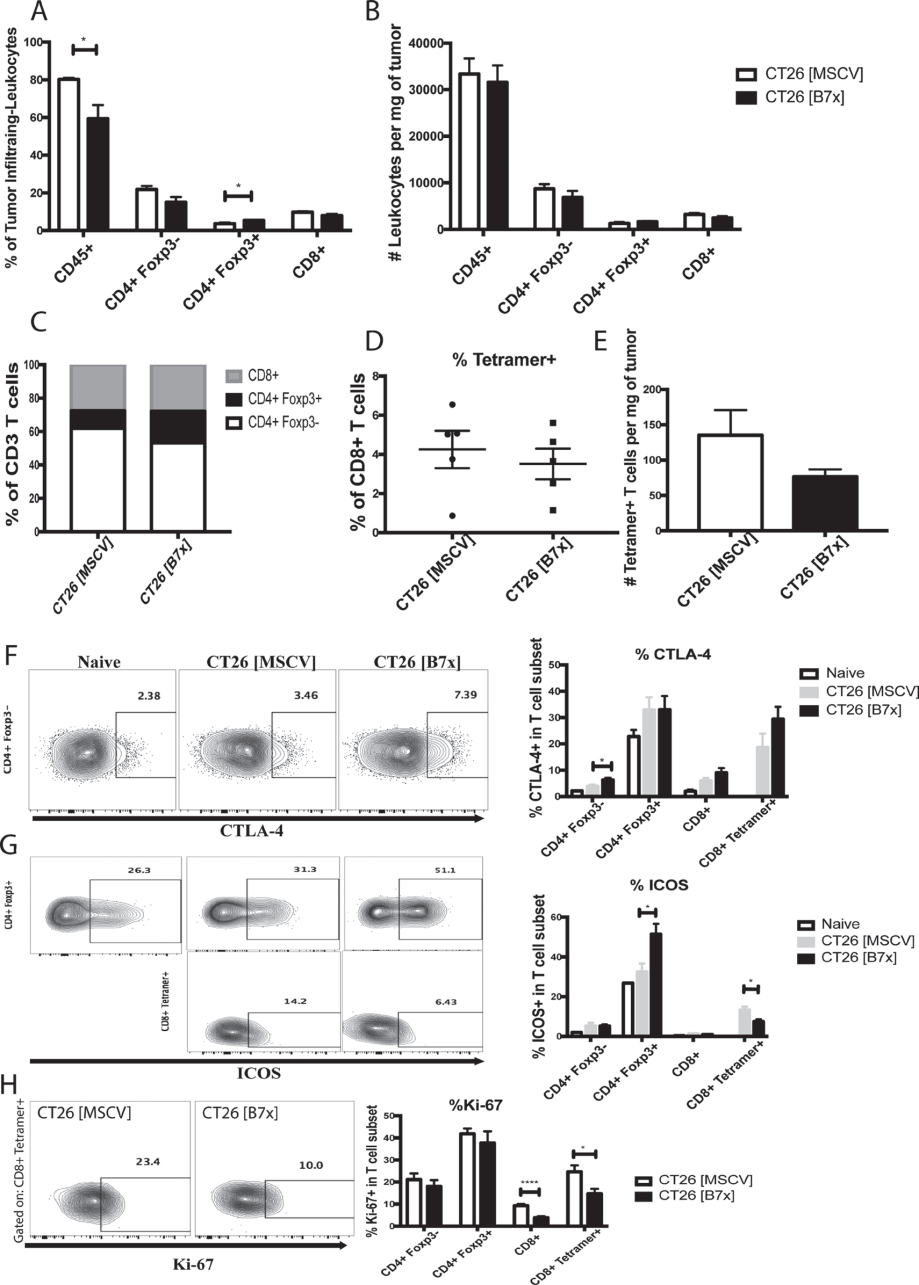
B7x increases percentage of T_regs_ and decreases ICOS expression and proliferation in antigen-specific CD8 T cells (**A**) Percent analysis of CD45^+^, CD4^+^ Foxp3^-^, CD4^+^ Foxp3^+^, and CD8^+^ T cells respectively in CT26 [MSCV] and CT26 [B7x] tumor bearing lungs approximately 17 days post i.v. injection. (**B**) Analysis of CD45^+^, CD4^+^ Foxp3^-^, CD4^+^ Foxp3^+^, and CD8^+^ T cells were quantified and analyzed per mg of tumor tissue 17 days following i.v. tumor injection. (**C**) Graphical depiction of the change in lymphocyte composition between two groups of mice. (**D**) Percent analysis of tetramer^+^ CD8^+^ T cells between CT26 [MSCV] and CT26 [B7x] 17 days post i.v. injection. (**E**) Analysis of tetramer^+^ CD8^+^ T cells were quantified and analyzed per mg of tumor tissue 17 days following i.v. tumor injection. (**F**–**H**) Quantification in the expression of CTLA-4, ICOS, and Ki-67 on T cell subsets in two groups of mice 17 days post i.v. tumor injection. Data are representative of three independent experiments. *p < 0.05, ****p < .0001. Error bars represent SEM.

After our studies demonstrated that B7x increased tumor metastases, we next sought out to dissect the immunological mechanisms causing the acceleration in disease. Following digestion of tumors we evaluated the composition and characteristics of tumor infiltrating lymphocytes (TILs) between both groups of mice seventeen days following tumor injection. The CT26 [B7x] group had significant decreases in the percentage of all CD45 positive cells found in the tumor milieu compared to control mice (Figure 3A). Upon further inspection of the TILs, though significance was not reached, it was found that B7x did cause a trend for decreasing percentages and numbers of CD4 and CD8 T cells (Figure 3A and 3B). However, the most significant observation was the dramatic increase in CD4^+^Foxp3^+^ T cell (T_regs_) percentages in the CT26 [B7x] groups of mice (Figure 3A).

Though there was not a significant difference in the percentages of CD4 T_eff_ (CD4+Foxp3-), when assessing the phenotypic properties of these cells it was found that cells in the CT26 [B7x] group expressed much higher levels of the co-inhibitory molecule CTLA-4 (Figure 3F). Analysis of CT26-specific tetramer positive CD8^+^ T cells also showed no significant changes in percentages and total numbers of this subset between both groups of mice, though there was a trend for a decrease (Figure 3D and 3E). However, it was notable to observe that the tetramer positive cells in the CT26 [B7x] group had significantly lower levels of co-stimulatory ICOS expression and were proliferating at a much slower rate as indicated by Ki-67 (Figure 3G–3H). Furthermore, it was also interesting to observe that T_regs_ in the CT26 [B7x] group expressed much higher levels of ICOS expression compared to CT26 [MSCV] control mice (Figure 3G), supporting recent literature suggesting that ICOS^+^ T_regs_ are more suppressive and predict lower overall survival [32].

### B7x contributes to an increase in MDSCs and enhances the M2/M1 ratio of TAMs

**Figure 4:**
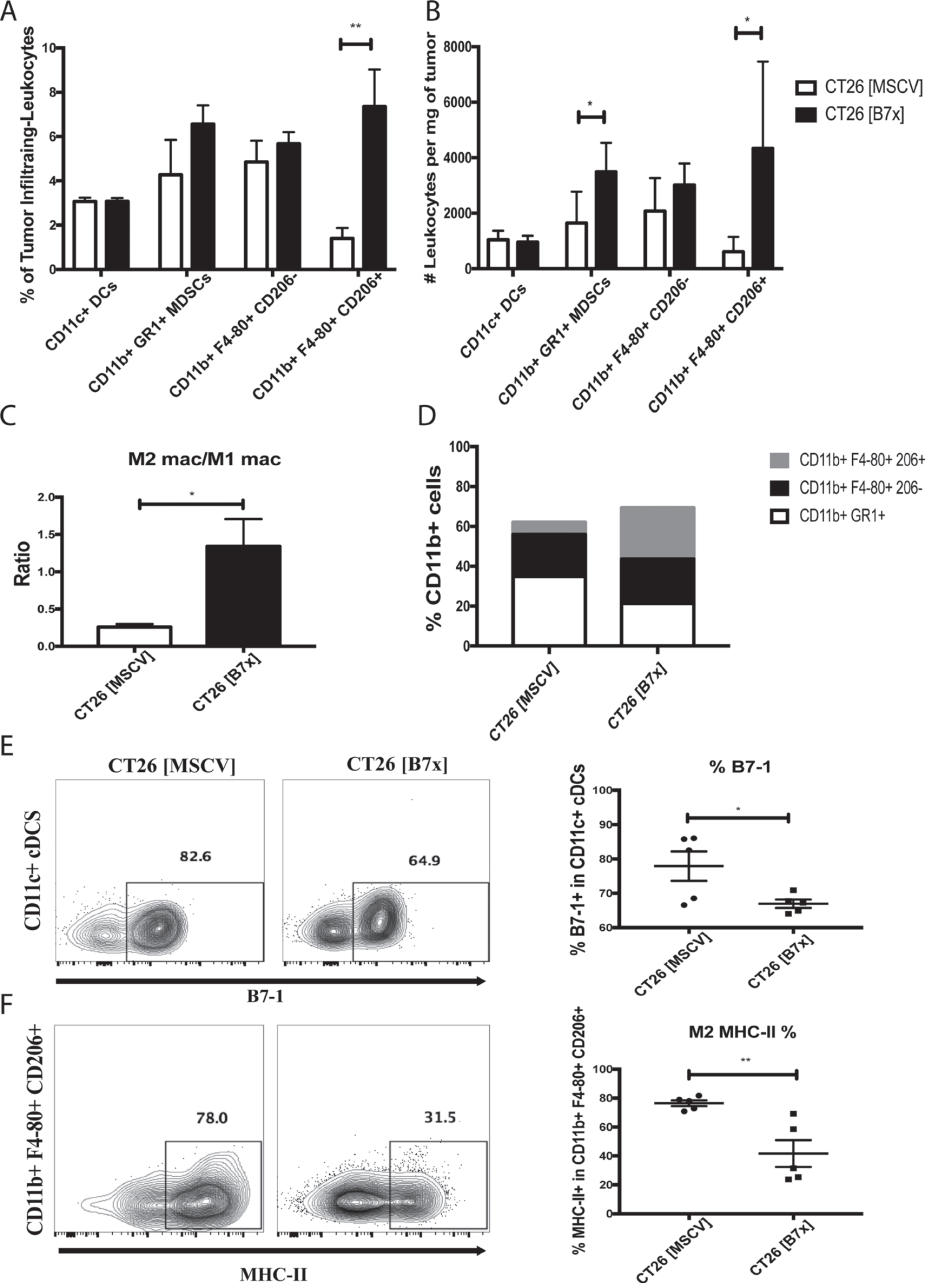
B7x promotes an increase in MDSCs and enhances M2 TAM polarization (**A**) Percent analysis of myeloid populations cDCs, MDSCs, M1 macrophages, and M2 TAMs respectively in CT26 [MSCV] and CT26 [B7x] tumor bearing lungs approximately 17 days post i.v. injection. (**B**) Analysis of the myeloid populations cDCs, MDSCs, M1 macrophages, and M2 TAMs were quantified and analyzed per mg of tumor tissue 17 days following i.v. tumor injection. (**C**) Cell numbers between M2 TAMs and M1 macrophages were divided and expressed as a ratio 17 days following tumor injections. (**D**) Graphical depiction highlighting the change in myeloid composition between groups of mice following 17 days post i.v. tumor injection. (**E**) Quantification of percent of B7-1 on CD11c^+^ cDCs and (**F**) MHC-II on M2 TAMs. Data are representative of three independent experiments. *p < 0.05, **p < 0.01. Error bars represent SEM.

Myeloid cells in the tumor microenvironment have proven to be critical mediators of tumor development; therefore we attempted to elucidate the composition and functional characteristics in several of these subsets in response to tumor-expressed B7x. To evaluate the impact of B7x on the intratumoral composition of these subsets we assessed percentages and counted the absolute numbers of CD11c^+^ conventional dendritic cells (cDCs), CD11b^+^ GR1^+^ myeloid derived suppressor cells (MDSCs), CD11b^+^ F4-80^+^ CD206^-^ M1 macrophages, and CD11b^+^ F4-80^+^ CD206^+^ M2 tumor associated macrophages (TAMs) and normalized these numbers by the tumor weight. We found that the composition of anti-tumoral cDCs and M1 macrophages were unaffected by tumor-expressed B7x between both groups of mice (Figure 4A–4B). However, when we evaluated pro-tumoral MDSCs and M2 TAMs, we observed a significant increase in the densities of these populations in the CT26 [B7x] group compared to CT26 [MSCV] controls (Figure 4B). This approximate seven-fold increase in TAMs as a result of B7x expression led to a five-fold increase in the M2 TAMs/M1 macrophage ratio, resulting in the greatest change in composition among the myeloid subset (Figure 4C–4D).

Despite the fact that B7x did not affect the density of cDCs between both groups of mice we did note that the expression of the costimulatory marker B7-1 was significantly reduced in the CT26 [B7x] group (Figure 4E). Along with this observation, we also saw a significant decrease in MHC-II expression in the TAMs in the CT26 [B7x] mice compared to CT26 [MSCV] controls (Figure 4F). Collectively this shows that B7x contributes to an increase in the densities of MDSCs and TAMs leading to greater tumor development and decreases their costimulatory and antigen-presentation capacities.

### B7x changes the balance between anti-tumor effector cells and immunosuppressive cells *in vivo*

**Figure 5:**
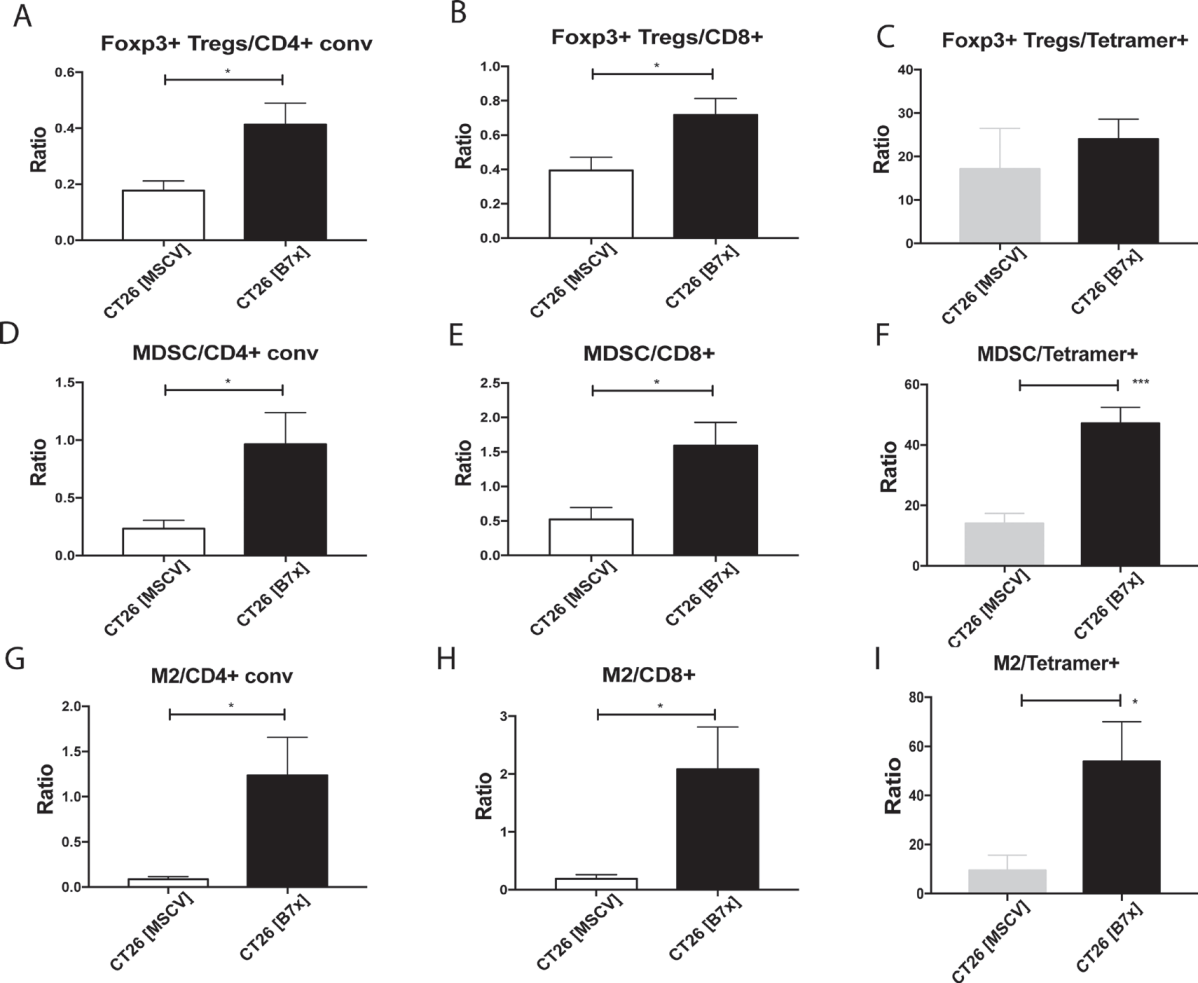
Tumor-expressed B7x generates highly immunosuppressive tumor microenvironment (**A**–**I**) Ratios of Foxp3^+^ T_regs_, MDSCs, and M2 TAMs to CD4^+^_conv,_ CD8^+^ T cells, and CD8^+^ Tetramer^+^ T cells were calculated from cell numbers isolated from tumor bearing lungs of CT26 [MSCV] or CT26 [B7x] 17 days following i.v. tumor injection. All data is representative from three independent experiments. *p < 0.05, ***p < .001. Error bars represent SEM.

Considering the significant increases in the immunosuppressive populations (T_regs_, MDSCs, and M2 TAMs) as a result of tumoral B7x expression, we decided to go further and obtain a snapshot of these subsets in relation to effector cells (CD4^+^, CD8^+^, and CD8^+^ Tetramer^+^ cells) to understand how the dynamic between these cells changes in the tumor microenvironment. Due to the increasing numbers of T_regs_ and decreasing numbers of CD4^+^ and CD8^+^ T cells, the ratio of T_regs_/CD4^+^ and T_regs_/CD8^+^ doubled while the ratio of T_regs_/ CD8^+^ Tetramer^+^ was unaffected (Figure 5A–5C). A similar pattern was also observed with MDSCs. Though, a four-fold increase was seen with the MDSC/CD4^+^ ratio and three-fold increases were equally observed with the MDSC/CD8^+^ and MDSC/CD8^+^ Tetramer^+^ ratios (Figure 5D–5F). Even larger changes were observed when we looked at the ratio of M2 TAMs to effector cells. B7x raised the intratumoral M2 TAMs/CD4^+^ ratio approximately five fold and both the M2 TAMs/CD8^+^ and M2 TAMs/CD8^+^ Tetramer^+^ ratios greater than ten-fold (Figure 5G–5I). Overall, this data shows that B7x promotes an enhanced ratio of immunosuppressive cells to effector cells and shifts the tumor microenvironment to a more pro-tumorigenic state.

### B7x promotes T cell exhaustion in antigen-specific CD8 T cells and inhibits pro-inflammatory cytokine secretion in CD4 and CD8 T cells

**Figure 6:**
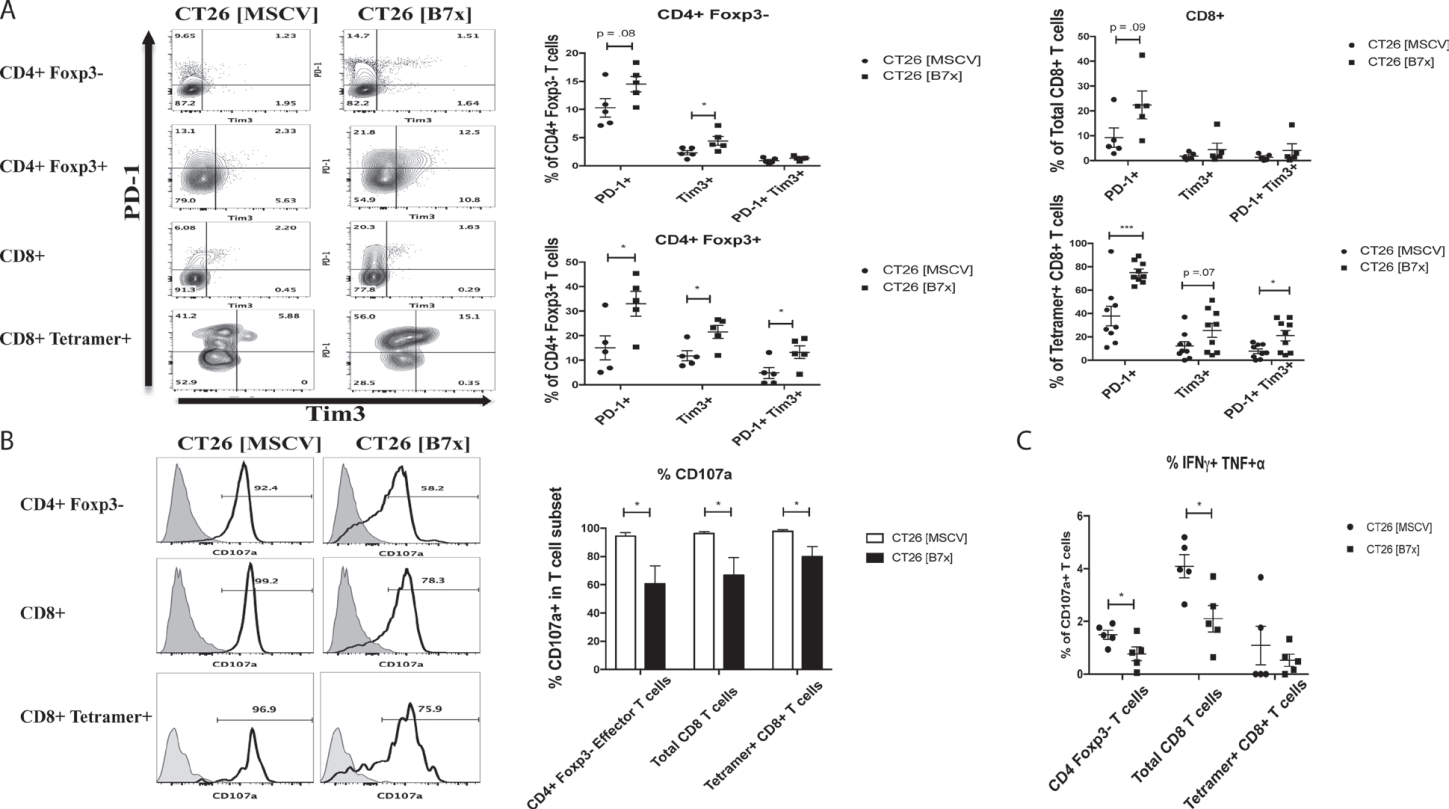
B7x influences antigen-specific T cell exhaustion and inhibits co-expression of pro-inflammatory cytokines in CD4 and CD8 T cells (**A**) T cell subsets were isolated from CT26 [MSCV] and CT26 [B7x] lungs 17 days post i.v. injection and stained for the markers indicative of exhaustion PD-1 and Tim3. Representative FACS plots of the frequencies are shown (left) and quantification of the percentages from each subset shown (right). (**B**) Immune infiltrates of the lung were harvested from tumor bearing lungs 17 days following i.v. injection and stimulated with PMA/Ionomycin for approximately 5 hours and stained for CD107a expression and (**C**) the co-expression of IFNγ and TNFα. Representative FACS plots of CD107a expression (B) are shown (left) and frequencies quantified (right). (C) Percentages of T cells are graphed from each effector subset. All data is representative from three independent experiments. *p < 0.05, ***p < .001. Error bars represent SEM.

Given the overwhelming numbers of immunosuppressive cells in the context of our experimental metastasis tumor model, particularly in the presence of B7x, we further investigated the effects of CT26 [B7x] on the function of TILs when compared to their CT26 [MSCV] counterparts. T cell exhaustion is a state of dysfunction with a loss of effector function marked by an increase in PD-1 and Tim3 expression observed in the context of chronic infection and cancer [33]. So we examined the expression of these two surface markers on TILs to determine the role B7x may have in influencing this exhaustive state. Though we didn’t see a change in the co-expression of PD-1 and Tim3 on CD4_eff_ T cells, there was a trend for an increase in the expression of PD-1^+^ alone and a significant increase in Tim3^+^ alone expression in the CT26 [B7x] group compared to the control group (Figure 6A). However, when we looked at CD8^+^ Tetramer^+^ T cells we did observe a three-fold increase in PD-1^+^ Tim3^+^ co-expression in the CT26 [B7x] group (Figure 6A) suggesting that B7x may play a role in the development of exhausted antigen-specific T cells. Interestingly, this approximate same three-fold increase in T cell exhaustion observed in CT26 specific CD8^+^ Tetramer^+^ T cells, was also observed in T_regs_ (Figure 6A) further supporting the capacity of B7x to contribute to T cell exhaustion.

As a consequence of this heightened T cell exhaustion, cytolyitc activity of CD4_eff_, total CD8, and CD8^+^ Tetramer^+^ was significantly decreased as measured by CD107a expression in the CT26 [B7x] group (Figure 6B). Additionally, when assessing further function of CD107a^+^ TILs between both groups of mice, we observed that B7x caused a significant decrease in polyfunctional CD4_eff_ and total CD8 T cells when looking at IFNγ and TNFα co-secretion (Figure 6C). Collectively, these observations demonstrate that tumor-expressed B7x inhibits the levels of tumor reactive helper and cytotoxic T cells in the tumor, which may contribute to tumor promotion.

### B7x does not directly interact with Neuropilin-1

**Figure 7:**
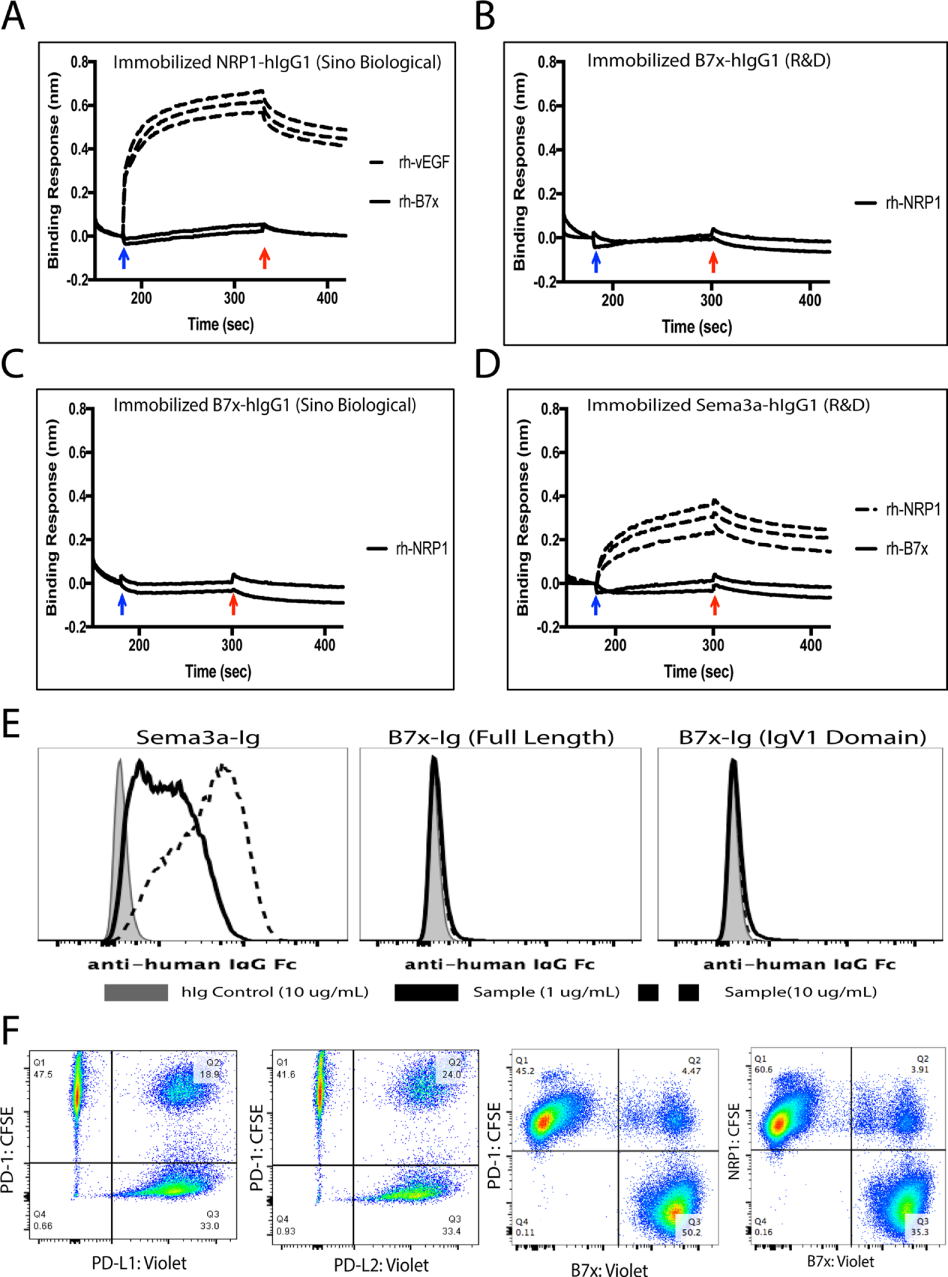
B7x does not directly interact with Neuropilin-1 (**A**) Human NRP1-hIgG1 (Sino Biological) was immobilized on sensors. Recombinant human vEGF (Peprotech) from concentrations of 0.025 mg/mL, 0.05 mg/mL and 0.1 mg/mL were challenged. The responses of vEGF were concentration dependent (dashed lines). Recombinant human B7x (R&D) was not responsive even at the concentrations of 0.25 mg/mL and 0.5 mg/mL (solid curve). (**B**) Human B7x-hIgG1 (R&D) was immobilized and recombinant human NRP1 (R&D) at the concentrations of 0.25 mg/mL and 0.5 mg/mL were challenged. (**C**) Human B7x-hIgG1 (Sino Bilogical) was immobilized and recombinant human NRP1 (R&D) at the concentrations of 0.25 mg/mL and 0.5 mg/mL were challenged. (**D**) Human Sema3a-hIgG1 (R&D) was immobilized on sensors. Rh-NRP1 (R&D) from concentrations of 0.025 mg/mL, 0.05 mg/mL and 0.1 mg/mL were challenged. The responses of vEGF were concentration dependent (dashed lines). Recombinant human B7x (R&D) was not responsive even at the concentrations of 0.25 mg/mL and 0.5 mg/mL (solid curve). The blue and red arrows indicate the starts of the association and dissociation, respectively. (**E**) 3T3 cells expressing Neuropilin-1 were incubated with either 10 ug/mL hIg control (shaded), 1 ug/mL (solid black line) or 10 ug/mL Sema3a-hIgG1, B7x-Ig, or B7x-Ig (IgV1 domain) protein (dashed black line) and then stained with APC-conjugated anti-human IgG Fc antibody to assess binding activity by FACS. (**F**) Incubation of labeled PD-1 with its ligands PD-L1 and PD-L2 resulted in 18% and 24% co-localization. Confirmed non-binding partners, PD-1 and B7x, resulted in approximately 4% non-specific binding, while NRP1 and B7x incubation led to approximately 3% co-localization. Data are representative of two separate experiments.

Elucidating the full functional capacity of B7x has been limited until now because its cognate receptor has yet to be discovered, therefore efforts to find this ligand have been studied heavily. Recently a patent application suggested that B7x interacted with Neuropilin-1 (NRP1)[26], so we decided to evaluate the potential binding. To determine the possible interaction between NRP1 and B7x, we carried out Bio-Layer Interferometry (BLI) assay. In these experiments, the human NRP1 protein was immobilized on the biosensor surface and challenged with various concentrations of vEGF and human B7x protein, respectively (Figure 7A). The responses of vEGF against immobilized NRP1 were significant and dose dependent. However, the responses of B7x against NRP-1 were insignificant and not dose dependent, suggesting no observable interactions between human NRP1 and B7x proteins (Figure 7A). We also immobilized human B7x protein and challenged against recombinant human NRP1. Both showed no response signals even at the concentration of 0.5 mg/mL NRP1 (Figure 7B–7C). We further tested if B7x interacts with Sema3a by immobilizing human Sema3a protein and then challenged with different concentrations of recombinant human NRP1 and human B7x, respectively (Figure 7D). NRP1 interacted with Sema3a with significant responses, whereas the recombinant human B7x did not interact with the Sema3a, indicating no observable interactions between these NRP1 and B7x proteins.

Moreover, we conducted a binding assay by FACS to confirm the results from our BLI analysis showing no signs of the interaction. We performed this by incubating various concentrations of commercial Sema3a-Ig, full length B7x-Ig, and the first immunoglobulin variable domain (IgV1) of B7x-Ig, fully functional protein we demonstrated to inhibit human T cell proliferation [34], with 3T3 cells engineered to stably express cell surface NRP1. The binding assay showed that Sema3a-Ig bound to the 3T3 cells expressing NRP1 in a dose dependent manner, whereas full length B7x-Ig and B7x-IgV1-Ig did not bind to the NRP1-expressing cells at all (Figure 7E). Furthermore, we conducted an intercellular conjugate assay that effectively illustrated the lack of binding between B7x and NRP1. Assay results highlighted potential binding was similar or lower to the negative control, B7x and PD-1, family members known to have no binding capacity (Figure 7F). This provided more evidence to support the fact that B7x does not directly interact with NRP1.

## DISCUSSION

One of the most striking features of B7x is that its mRNA expression is significantly higher in peripheral non-lymphoid tissues compared to lymphoid tissues, a sharp contrast from other members of the B7 ligand and CD28 receptor family [9]. Despite the features of B7x mRNA expression in peripheral non-lymphoid tissues, endogenous expression of the protein in normal human tissues is quite limited. However, analysis of cancerous tissues reveals an overexpression of B7x in a high proportion of patients’ tumors in various malignancies [35] with inductive stimuli for the expression not clear. Current literature suggests that the secretion of cytokines IL-10 and IFNγ from TAMs can up-regulate B7x on lung cancer and hematological malignancies like Non-Hodgkin Lymphoma (NHL) [27, 28], however our studies did not confirm this after assaying several different human and murine tumors. Our aim was to evaluate changes in surface expression of B7x, but reports have demonstrated intracellular cytoplasmic and nuclear expression of B7x that have been affected under hypoxic conditions [36, 37]. Therefore, pro-inflammatory and anti-inflammatory cytokine may have an impact on intracellular B7x. Additionally, a combination of several cytokines, typically found in the tumor microenvironment, may be needed to consistently induce B7x expression.

Due to the fact endogenous B7x was not found or induced on any murine tumor cell lines we generated a colonic murine tumor cell line to stably express B7x. Following injection of the CT26 [B7x] tumor cells i.v., we demonstrated that B7x accelerated the development of pulmonary metastases compared to the CT26 [MSCV] controls. This is in accord with a majority of current clinical studies highlighting that tumor-expressed B7x associates with a poor clinical outcome [21, 38, 39].

In-depth examination of the immune cell infiltrate in our model revealed interesting results that we have yet to see be reported with respect to tumor-expressed B7x. The increase of CD4^+^ Foxp3^+^ T_regs_ in CT26 [B7x] mice compared to CT26 [MSCV] controls, were consistent with previous studies in B7x knockout mice compared to wild-type mice and B7x blockade [22, 24]. However the description of B7x contributing to their increased expression of ICOS was a novel observation. This evidence contributed to the thought that B7x may influence the development of a more functionally suppressive T_reg_ subset that predicts reduced survival [32]. On the other hand, we also found that tumoral B7x expression caused a marked reduction of ICOS on antigen-specific CD8^+^ T cells, a marker indicative of effector function in T cell subsets [40, 41]. Furthermore, we also show for the first time the increased expression of another CD28 receptor family member, coinhibitory CTLA-4, on CD4^+^ T_eff_ cells.

With regard to the innate immune system, the increased content of MDSCs in the CT26 [B7x] group of mice strengthened the validity of current literature supporting that host B7x regulates MDSC proliferation and function in the tumor microenvironment [22, 23]. MDSCs are critical in suppressing the adaptive and innate immune system during tumor progression by secreting immunosuppressive factors such as arginase and IL-10 [42], so to observe this consistent finding in our tumor-expressed B7x model was interesting. The most striking observation we saw with respect to innate immunity was the striking polarization of TAMs from an M1 phenotype to an M2 phenotype in CT26 [B7x] mice versus CT26 [MSCV] controls. M2 TAMs constitute a significant portion of the tumor-infiltrating immune cells and contribute to cancer growth, metastases, and poor patient prognosis [43]. Therefore it is very interesting that our data implicated the role of B7x in regulating the density and phenotype of this population and anticipate looking deeper into this in the future. Collectively, this rise in immunosuppressive immune cells in relation to effector cells showed that B7x was able to inhibit the proliferation of antigen-specific CD8^+^ T cells.

Further analysis of T cells in our model highlighted the novelty in B7x playing a role in promoting a significant increase in T cell exhaustion in antigen-specific CD8^+^ T cells when looking at PD-1^+^ Tim3^+^ co-expression. In line with this T cell exhaustion, we also saw that antigen-specific CD8^+^ T cells in the CT26 [B7x] group had much lower cytolytic activity compared to CT26 [MSCV] mice evidenced by CD107a expression, though this did not lead to a significant decrease in the co-expression of IFNγ and TNFα. In addition, tumor-expressed B7x increased an exhaustive phenotype in T_regs_. This is an interesting observation because recent studies have shown that the co-expression of Tim3 and PD-1 on T_regs_ correlates with not only tumor size and immunosuppression, but that it also directly associates with poor clinical prognosis [44, 45]. Our results illustrate this potential link and provide evidence for B7x being a contributing factor.

NRP1 is a molecule expressed highly on regulatory T cells and M2 macrophages and confers enhanced immunosuppressive activity that binds highly to Sema3a and vEGF [46, 47]. Therefore, we were intrigued at literature reporting a potential interaction between B7x and NRP1[26], given the B7x associated rise in T_regs_ and M2 TAMs found in our tumor model. However, after experiments of both BLI assay and FACS, no evidence was found for the direct interaction between B7x and NRP1. Though this does not rule out the possibility of NRP1 binding indirectly to B7x with other accessory proteins as a complex. In summary, our results, utilizing a colonic pulmonary metastasis model, illustrate a phenotypic description of the immune-evasive role of B7x in promoting tumor metastases, while highlighting new observations within both the innate and adaptive immune system. However, further studies are necessary to precisely elucidate the relationship between tumor-expressed B7x and its still unknown cognate receptor(s) in the tumor microenvironment.

## MATERIALS AND METHODS

### Animals

Six to eight week old BALB/c mice were purchased from the National Cancer Institute and Charles River Laboratories. Mice were housed in a specific-pathogen-free facility at the Albert Einstein College of Medicine, and experiments were performed with respect to Institutional Animal Care and Use Committee guidelines.

### Cell lines

The human cancer cell lines MDA MB 468 (breast), SKBR3 (breast), U2OS (osteosarcoma) and OVCAR4 (ovarian), and murine cancer cell lines CT26 (colon) and MC38 (colon) were cultured in RPMI-1640 containing 10% fetal bovine serum (FBS), 100 u/mL penicillin, 2 mM L-glutamine, 1% nonessential amino acids (NEAA), and 1 mM sodium pyruvate. Murine cancer cell lines Lewis Lung Carcinoma (LLC) and Panc02 (pancreas) along with the mouse fibroblast 3T3 cell line were maintained in DMEM containing 10% FBS, 100 u/mL penicillin, 2 mM L-glutamine, 1% NEAA, and 1 mM sodium pyruvate. Murine-B7x/MSCV and human-Neuropilin-1/MSCV vectors were used to generate retrovirus to transfect CT26 and 3T3 cells respectively to establish stable CT26/B7x and 3T3/Neuropilin-1 cell lines. B7x and Neuropilin-1 protein expression was confirmed by FACS analysis. In addition, CT26 was transfected with MSCV vector alone as a control CT26/MSCV.

### Cell stimulation

For *in vitro* stimulation, 2 × 10^5^ human or murine cancer cell lines were plated in a six well plate to adhere overnight. On the subsequent day, cells were stimulated with either human or mouse IL-10, TNFα, or IFN-γ (Tonbo Biosciences) at 50 ng/mL for 48 h and evaluated for expression of MHC-I, MHC-II, PD-L1, and B7x by FACS.

### Cell proliferation

Control CT26 [MSCV] and CT26 [B7x] cells (2 × 10^3^) were plated in triplicates in a 96 well plate for 24-72 h in complete media. *In vitro* cellular proliferation was determined by the Cell Counting Kit-SK assay (Dojindo Laboratories) and plates read at 450 nm.

### Animal tumor studies

A total of 10^5^ CT26 [MSCV] or CT26 [B7x] were intravenously (i.v.) injected into the tail vein of wild-type BALB/c mice in 200 uL of PBS to induce the pulmonary experimental metastasis model. Approximately seventeen days following injection, mice were sacrificed to assess tumor burden or evaluate the immune cell infiltrate. Lung metastasis quantification was performed by intratracheally injecting 5 mLs of 15% India Ink (Sigma) into each lung, then fixing and staining the lungs overnight in Fekete’s solution. Tumor nodules were enumerated using a dissecting microscope. For immune cell isolation, mice were anesthetized and perfused with PBS, tumors were then excised, weighed, and handled precisely as instructed to by the protocol accompanying a Mouse Tumor Dissociation Kit (Miltenyi Biotec) and processed by a gentleMACS Dissociator (Miltenyi Biotec). Following tumor digestion, cell suspensions underwent RBC lysis (Tonbo Biosciences) and filtered into FACS buffer (1% BSA in PBS) for downstream experiments.

### Flow cytometry: surface staining

Murine cells were incubated with a Ghost Dye Red 780 (Tonbo Biosciences) viability marker and anti-mouse CD16/32 (Tonbo Biosciences) in FACS buffer for 30 minutes on ice to block murine Fc receptors. Cells were then stained with the following fluorophore-conjugated anti-mouse monoclonal antibodies: CD45, CD3, CD4, CD8, ICOS [C398.4A], CD11c, CD11b, GR1 [RB6-8C5], F4-80, CD206 [C068C2], B7-1, MHC-I [34-1-2S & 28-8-6], MHC-II [M5/114.15.2], PD-1, Tim3, PD-L1, and B7x [HMH4-5G1] (all from Biolegend); and SPSYVYHQF/H-2L^d^ Alexa-647 conjugated tetramer [NIH Tetramer Core Facility]. All antibodies were stained for an additional 45 minutes on ice. Human cells were incubated with the Ghost Dye Red 780 viability marker for 30 minutes on ice and immediately stained with the following fluorophore-conjugated anti-human monoclonal antibodies: MHC-I, MHC-II, PD-L1, and B7x [MIH43] (all from Biolegend) on ice for 45 minutes.

### Flow cytometry: intracellular staining

Following cell surface staining cells were permeabilized using the Foxp3 staining buffer set (eBioscience) and stained with the following fluorophore -conjugated anti-mouse antibodies: Foxp3 [FJK-16s] (eBioscience); CTLA-4 and Ki-67 (Biolegend).

### Intracellular cytokine staining

Single cell suspensions were stimulated at 37°C for ∼5 h with Cell Stimulation Cocktail (Tonbo Biosciences) in complete RPMI media. Following the incubation period, cells were washed and stained with the Ghost Dye Red 780 viability marker, blocked for Fc receptors and surface stained for CD45, CD3, CD4, CD8, and Tetramer for 45 minutes on ice. Subsequently they were then permeabilized and stained with Foxp3, CD107a, IFNγ, and TNFα for 45 minutes on ice.

### Flow cytometry acquisition and analysis

Samples were acquired on a LSR II or LSR II Yelow (BD Biosciences) and analyzed with FlowJo software. Using forward scatter, side scatter, and the viability dye, live cells were gated on. Doublets were then excluded and the singlets gated on CD45^+^ cells and other additional lineage markers.

### Bio-layer interferometry assay

Measurement of the interactions were carried out by the BLItz system (ForteBio) as described [48]. Briefly, the anti-human IgG Fc capture sensors (ForteBio) were hydrated for 15 minutes in H_2_O solution and then dipped into the human IgG fusion protein solution at the concentration of 25 ug/mL for 2 minutes to immobilize the proteins on the sensor. The resulting sensors loaded with the IgG fusion proteins were dipped in the 1 × Kinetic Buffer (ForteBio) for 30 seconds to generate the baseline. The sensors were then challenged with protein analytes to measure the association curves. The sensors were then moved back into 1 × Kinetic Buffer (ForteBio) to measure the dissociation curves. The immobilized human IgG fusion proteins were purchased from R&D systems and Sino Biological Inc. Recombinant human vEGF was purchased from Peprotech. Recombinant human B7x and recombinant human NRP1 were purchased from R&D systems

### Cell-fusion protein binding assay

3T3 cells expressing full length human Neuropilin-1 (1 × 10^5^) were washed and re-suspended in 100 uL of FACS buffer in a 96 well plate on ice. Then either human-IgG1, Semaphorin3a-hIgG1 (company), full length human B7x-IgG1 (company), or the IgV1 domain of human B7x (B7x-IgV1-IgG1) was added to the cell suspension at various concentrations and incubated for 45 minutes on ice. The cell and protein mixture was then washed with FACS buffer and stained with an anti-human IgG Fc antibody (Biolegend) for 45 minutes on ice. The suspension was washed twice and analyzed by FACS. All fusion proteins were obtained by R&D systems except for B7x-IgV1-IgG1, which was generated as previously described [24].

### Intercellular conjugate assay

Cells of interest are resuspended in cold FACS buffer and labeled according to the manufacturers protocol: CFSE (2–3 uM) and Violet Cell Trace (2–3 uM). The labeled cells (2 × 10^5^) were then plated in a 96-well plate round bottom plate in 100 uL of FACS buffer and incubated with the same quantity in number of the other cells of interest. Cells have to be labeled with different cell tracking dyes that have no spectral overlap. The cell mixture was then incubated in a 37 degrees Celsius incubator for 45 minutes undisturbed and spun at 1500 rpm for 3 minutes at 4 degrees Celsius. The cells were then washed with 200 uL of FACS buffer and submitted for FACS analysis and acquisition.

### Statistics

Statistical analysis was performed with the Prism software (GraphPad) using the unpaired Student *t* test or the log-rank test for survival studies. For all statistical tests conducted, significance was accepted when *p* < 0.05. Data was expressed as mean ± SEM unless indicated otherwise.
